# Around the clock: gradient shape and noise impact the evolution of oscillatory segmentation dynamics

**DOI:** 10.1186/s13227-018-0113-2

**Published:** 2018-12-10

**Authors:** Renske M. A. Vroomans, Paulien Hogeweg, Kirsten H. W. J. ten Tusscher

**Affiliations:** 1 Centre of Excellence in Experimental and Computational Developmental Biology, Institute of Biotechnology, University of Helsinki, Viikinkaari 5, 00790 Helsinki, Finland; 2 Theoretical Biology, Utrecht University, Padualaan 8, 3584CH Utrecht, Netherlands

**Keywords:** Segmentation, Travelling waves, Oscillations, Complexity, Robustness

## Abstract

**Background:**

Segmentation, the subdivision of the major body axis into repeated elements, is considered one of the major evolutionary innovations in bilaterian animals. In all three segmented animal clades, the predominant segmentation mechanism is sequential segmentation, where segments are generated one by one in anterior–posterior order from a posterior undifferentiated zone. In vertebrates and arthropods, sequential segmentation is thought to arise from a clock-and-wavefront-type mechanism, where oscillations in the posterior growth zone are transformed into a segmental prepattern in the anterior by a receding wavefront. Previous evo-devo simulation studies have demonstrated that this segmentation type repeatedly arises, supporting the idea of parallel evolutionary origins in these animal clades. Sequential segmentation has been studied most extensively in vertebrates, where travelling waves have been observed that reflect the slowing down of oscillations prior to their cessation and where these oscillations involve a highly complex regulatory network. It is currently unclear under which conditions this oscillator complexity and slowing should be expected to evolve, how they are related and to what extent similar properties should be expected for sequential segmentation in other animal species.

**Results:**

To investigate these questions, we extend a previously developed computational model for the evolution of segmentation. We vary the slope of the posterior morphogen gradient and the strength of gene expression noise. We find that compared to a shallow gradient, a steep morphogen gradient allows for faster evolution and evolved oscillator networks are simpler. Furthermore, under steep gradients, damped oscillators often evolve, whereas shallow gradients appear to require persistent oscillators which are regularly accompanied by travelling waves, indicative of a frequency gradient. We show that gene expression noise increases the likelihood of evolving persistent oscillators under steep gradients and of evolving frequency gradients under shallow gradients. Surprisingly, we find that the evolutions of oscillator complexity and travelling waves are not correlated, suggesting that these properties may have evolved separately.

**Conclusions:**

Based on our findings, we suggest that travelling waves may have evolved in response to shallow morphogen gradients and gene expression noise. These two factors may thus also be responsible for the observed differences between different species within both the arthropod and chordate phyla.

**Electronic supplementary material:**

The online version of this article (10.1186/s13227-018-0113-2) contains supplementary material, which is available to authorized users.

## Background

Evolutionary developmental biology aims to understand how the developmental patterning mechanisms evolved that shape complex organisms. It also seeks to answer why evolution favours certain patterning mechanisms over alternative, theoretically possible, mechanisms, and whether and how these mechanisms can change into one another. Segmentation, the division of the body axis into repeated units, is considered a major evolutionary innovation and has been intensely studied on the level of the developmental mechanism and from an evolutionary perspective. Within the animal clade, there are three lineages with a clearly segmented organization: annelid worms, arthropods and chordates [1, 2]. There are both striking similarities and differences in the segmentation mechanism used by different species both between and within clades, making segmentation an ideal subject for evo-devo questions.

In most segmented animals, segments are generated from a posterior growth zone and laid down in a regular anterior–posterior sequence. Sequential segmentation has been studied in most detail in vertebrates, where somites emanate sequentially from a posterior undifferentiated zone, the presomitic mesoderm (PSM), in which oscillatory gene expression occurs. A wavefront retreating across the PSM transforms this oscillatory gene expression into a spatially repeated pattern of segments (for review, see, e.g. [3]). Most arthropods appear to deploy a similar sequential segmentation mode although the molecular details underlying oscillations and the transformation to segments are still incompletely understood [4]. In addition to sequentially segmenting arthropods, amongst which the so-called short germband insects, also intermediate and long germband insects exist. These two types of insects pattern, respectively, their anterior segments or all their segments simultaneously, using a different developmental mechanism. While the segmentation process in annelids is also sequential, cell lineages with a different future fate are specified before segmentation through stereotyped divisions and appear to undergo distinct parallel sequential segmentation processes before fusing into segments [5].

Previous evo-devo simulation studies demonstrated that oscillation-driven sequential segmentation readily evolves out of an initial random gene regulatory network (not structured by prior evolution). References [6–10], provided that a posterior signalling centre has previously evolved [10]. These studies also showed that this type of segmentation mechanism should be expected to evolve due to its higher robustness and its greater ability to flexibly adjust segment numbers relative to alternative strategies. However, thus far, the potential conditions and selective pressures that cause differences in the more detailed aspects of sequential segmentation have remained unresolved.

In vertebrates, the oscillatory nature of segment patterning was originally discovered from the observation of waves of gene expression traversing the unsegmented tissue [11]. These gene expression waves were shown to arise independently of cell–cell contact [11] and instead result from the gradual slowing down of oscillations before they arrest into segments [11–14]. Apart from these so-called kinematic waves, vertebrate segmentation is characterized by a complex regulatory network consisting of three coupled oscillator motifs involving the FGF, Wnt and Delta-Notch signalling pathways [15–17]. Kinematic waves have also been observed in sequentially segmenting arthropods, for example the centipede Strigamia [18, 19]. It has been suggested that oscillator slowing is a crucial part of the mechanism underlying the transition from oscillatory gene expression to segments [20, 21] or instead that it is an emergent property (a “side effect”) of cell–cell signalling [22]. Additionally, it has been hypothesized that travelling waves enhance the robustness of the segmentation process [23]. Intriguingly, in both the chordate and arthropod lineages, variation exists in the extent of these travelling waves and the length of the undifferentiated region between the growth zone proper and the last-formed segment. For instance, in Amphioxus (a non-vertebrate chordate), segments are formed directly anterior to a small posterior zone, and no travelling wave dynamics have been reported thus far [24]. On a similar note, in the short-germ beetle Tribolium, travelling waves have been reported but the relative distance they travel before halting appears to be shorter than, for example, in Strigamia [4, 18].

When considering the genetic composition of the oscillator, Amphioxus does not seem to require FGF and also RA appears to be less involved than in vertebrates [25, 26]. This could potentially indicate a simpler oscillator architecture. Similarly, in Tribolium, so far only a simple negative feedback loop of pair-rule genes has been shown to underlie segment oscillations in the trunk [27], while in other sequentially segmenting insects this loop has not been identified, and possibly more complex mechanisms are at play [28]. One tempting possibility could thus be that more complex oscillators are correlated with and potentially responsible for more extensive kinematic waves. Alternatively, oscillator complexity may be related to mutational and developmental robustness and occur independent of kinematic waves. Finally, apparent oscillator simplicity in, for example, Amphioxus and Tribolium may merely reflect a lack of available data, and as a consequence the relation between kinematic waves and oscillator complexity is currently unclear.

Here, we applied an evo-devo modelling framework to investigate under which conditions complex oscillator networks and travelling oscillator waves are likely to evolve, and to what extent they co-occur. Based on the observations outlined above, we speculate that differences in travelling wave dynamics could arise from the difference in relative size of the non-segmented zone between species, which are likely caused by differences in morphogen gradient lengths and slopes. We therefore vary the rate of morphogen decay to test the impact of gradient length scale and slope on the type of oscillatory segmentation that evolves. Since it is unclear to what extent oscillator complexity is necessary for either kinematic waves or developmental robustness, we also investigate the influence of gene expression noise on the phenotype resulting from evolution. To analyse large numbers of simulations more efficiently, we build an automated analysis pipeline to assess oscillator complexity and the occurrence of travelling waves.

We find that shallow, long morphogen gradients often lead to the evolution of persistent oscillations, travelling waves and complex networks. In contrast, simulations with steep, short morphogen gradients resulted in slightly simpler networks and more often produced damped oscillators, while sequential segmentation evolved faster. Damped oscillators are more sensitive to perturbations and less easily allow for evolution of longer body axes containing more segments.

Interestingly, gene expression noise increased the fraction of persistent oscillators under a steep gradient and also increased the fraction of travelling wave oscillators for both shallow and steep gradients. This suggests that in our model, evolution of oscillator slowing is enhanced by (indirect) selection for robustness. Surprisingly, we found that gene regulatory network complexity and oscillator slowing, both typical for vertebrate somitogenesis, did not evolve in a strongly correlated manner in our model. This implies that these properties may evolve separately.

## Methods

### The model

#### General set-up

**Fig. 1:**
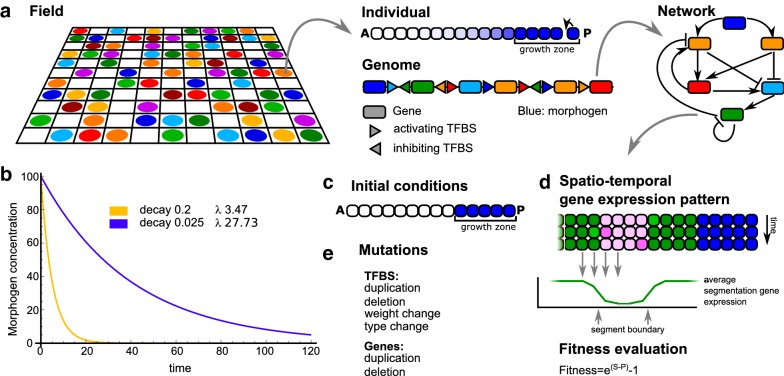
Overview of the model. **a** The developing organisms live on a 2D lattice. Each individual organism consists of a row of cells, of which the posterior-most cell divides at regular intervals. Within the growth zone, the morphogen (in blue) is maintained at a high concentration; it decays in cells outside of this zone. The genome of the organism codes for a network of regulatory interactions, which determines the spatio-temporal dynamics of the proteins within each cell (see **d**). **b** The gradients resulting from the different morphogen decay rates (**d**) used in our simulations. The lambda indicates the position (or time) at which the morphogen concentration is half-maximal, i.e. 50: $\lambda ={\mathrm{ln}}(2)/d$. **c** The initial conditions for each new individual at the start of its development. There is a growth zone with high morphogen, and a “head” region without morphogen. **d** At the end of development, the expression of the segmentation gene is averaged over a number of time steps, and from this the segment boundaries are determined. **e** The mutational operators acting on the genome

We use an individual-based model of a population of organisms evolving on a lattice, as has been applied before to evolution of segmentation and domains [8, 10] (Fig. 1a).

Each organism has a so-called pearls-on-a-string genome consisting of genes (encoding transcription factors) and upstream regulatory regions with transcription factor binding sites (TFBS) [29]. Organisms also have a highly simplified multicellular body consisting of a one-dimensional row of cells. Instead of starting at full length as in previous models (for review, see [9]), organisms start out small and grow during the course of their development. The organisms reproduce in a fitness-dependent fashion, with fitness dependent on the number of segments pre-patterned by the final gene expression pattern in the row of cells. Importantly, since we explicitly select for segments, our modelling approach can not help answer why body axis segmentation evolved. However, no selective pressure is exerted on how segments should be generated, so evolution is free to evolve any mechanism capable of generating segments. Therefore, we can use our model to investigate how certain conditions influence what types of segmentation mechanisms evolve.

#### Individuals

*Genome, network and genes* The genome codes for a gene regulatory network. The genes in the genome form the nodes of the network; the set of TFBS upstream of each gene in the genome dictate the incoming regulatory edges of the GRN (Fig. 1a). Outgoing edges follow from genes matching the type of the TFBS in front of another gene. The regulatory interactions between genes can be repressive (strength $-1$) or activating (strength 1). The network governs gene expression dynamics and subsequent protein levels. Gene expression is modelled with ordinary differential equations as shown in Eq. 1:

1 $$\begin{aligned} \frac{{\mathrm{d}}G_i}{{\mathrm{d}}t}={\mathrm{Max}}_{j=1}\left( \frac{A_j^n}{A_j^n+H^n}\right) *\Pi _{k=1}\left( \frac{H^n}{I_k^n+H^n} \right) *E-\delta *G_i \end{aligned}$$

Transcription of gene *i* is determined by the activating genes $A_j$ ($j=1\ldots l$), where the activator with the highest activating input (as given by $\frac{A_j^n}{A_j^n+H^n}$) determines the overall activation, resulting in a so-called activating OR gate. Repressive inputs $I_k$ ($k=1\ldots m$) are multiplied, resulting in a repressive AND gate (*l* and *m* are the total number of activating and repressing inputs for gene *i*). It should be noted that these choices are somewhat arbitrary, as for both activating and repressive TFs, AND as well as OR or even different types of integration have been reported. The main goal here is to incorporate at least partially the highly complex, nonlinear integration of TF inputs into gene expression levels. *E* is the maximum expression level; $\delta$ is the degradation rate; *H* is a Hill constant, the transcription factor concentration level at which half-maximal activation or repression occurs; and *n* is the Hill coefficient governing the steepness of the transition from low to high gene expression depending on transcription factor concentrations.

There are 16 types of genes, indicated with a number from 0 to 15.

*Gene 0 encodes the morphogen* It is not regulated by any of the other gene products, but instead is set to high expression in the cells of the growth zone, while decaying with a predefined rate in the rest of the embryo (Fig. 1b). We run simulations with either a large or a small morphogen decay rate, yielding a steep or a shallow morphogen gradient, respectively.

*Gene 5 encodes the segmentation protein*, whose final expression pattern after development determines the number of segments formed and hence the fitness of the organism.

*Gene expression noise* In a subset of simulations, we implemented gene expression noise as follows. First, we computed the expected gene expression rates based on the first part of Eq. 1. Next, we computed the actual gene expression rate by sampling from a Gaussian distribution around the expected gene expression rate. Specifically, we assume a Gaussian distribution with a mean equal to the computed expected gene expression rate $R_{\mathrm{expr}}$ ($\mu =R_{\mathrm{expr}}$, $\sigma =l*R_{\mathrm{epxr}}$), where *l* in the standard deviation $\sigma$ determines the overall level of noise (low: $l=0.07$, medium: $l=0.14$, high: $l=0.21$). Note that by scaling the standard deviation with the mean, the noise which is defined as the standard deviation divided by the mean, is kept constant independent of the mean gene expression rate. We avoid negative gene expression rates by capping any negative gene expression rates due to noise to zero: $R_{\mathrm{actual}}={\mathrm{Max}}(0,R_{\mathrm{expr}}+{\hbox {noise}})$.

*Developmental dynamics* Individuals start their development with a short row of 14 cells, where five cells form the primordial “growth zone” in which the morphogen concentration is high; in the remaining nine cells (the “head”), the morphogen is absent (Fig. 1c). The other genes have an expression level of 0 in all cells. This means that no gene expression will occur in the anterior-most nine cells. We ignore the developmental processes generating the head part of the body and their evolution and focus solely on the developmental processes governing formation of more posterior body parts and their evolutionary history. The posterior-most cell of the growth zone divides at regular intervals, pushing the other cells forward so that they eventually move out of this zone. Once a cell leaves the growth zone, the morphogen protein starts decaying. As a result, a gradient of the morphogen is formed due to the age difference of the cells (Fig. 1a, b). (The four cells in the growth zone that do not divide are there for cosmetic reasons; it makes it easier to see the dynamics in the growth zone on a time–space plot.) Throughout development, the concentrations of the other proteins (i.e. all except the morphogen protein) are updated according to the genetically specified network interactions (Eq. 1). The posterior cells stop dividing after 120 divisions (600 steps), after which developmental dynamics continue for another 600 time steps (see also Table 1) so that also the youngest cells reach a low morphogen concentration and can converge on a stable gene expression pattern.

**Table 1:** parameter values

Parameter	Values	Remarks
*General*		
Grid size	$30\times 30$	
Evolutionary time steps	10,000	
Death rate	0.5	
Initial # agents	50	
*Development*		
Developmental time steps	1200	The number of integration steps
Duration of division period	600	Divisions occur every five time steps
Duration of stabilization period	600	Period without divisions
Integration step size	0.2	Forward Euler integration
Morphogen decay rate	0.025 or 0.2	
Initial tissue size	14 cells	Of which nine form the head
*Gene and protein dynamics*		
Gene product decay rate	0.3	
Hill constant of the TFBS	60	
Gene transcription	100	
*Mutational dynamics*		
Nr of gene types	16	
Gene duplication	0.006	Note that with the gene, also its TFBS are duplicated
Gene deletion	0.009	
TFBS weight change	0.001	
TFBS type change	0.001	
TFBS duplication	0.0015	
TFBS deletion	0.004	
TFBS innovation	0.001	Spontaneous emergence of new TFBS
*Fitness*		
G: penalty per gene	0.0005	
T: penalty per TFBS	0.00005	
Control period	100 steps	Period over which gene expression stability is measured
U: expression variance penalty	0.1	Penalty per cell that has a variance in segmentation gene level > 5.0 during the control period

**Fig. 2:**
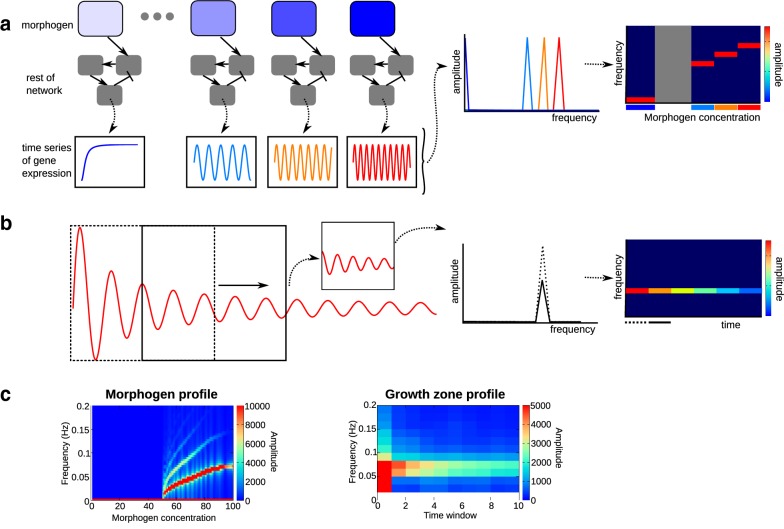
Explanation of the Fourier analysis procedure. **a** We run the evolved network for 1800 steps with several, fixed concentrations of the morphogen. For every gene, we take the Fourier transform of the temporal gene expression dynamics to find the gene’s oscillation frequency for that particular morphogen concentration. We plot the Fourier transform data of all concentrations together in one heat map, where the colour intensity represents the amplitude at every frequency for every concentration. See also **c** for a “real-life” example. **b** For the network run at the highest morphogen concentration (representing the growth zone), we also perform a sliding-window analysis: here, we take subsets of the time series generated as in **a** and apply the Fourier transform to every window to visualize the change in frequency and amplitude over time in the growth zone. The rest of the procedure is the same as in **a**. **c** Examples of frequency profiles from real simulations. The plots in the left column are generated as explained in **a**, and those on the right as in **b**

*Fitness evaluation* By the end of development, the expression pattern of the segmentation gene is evaluated to determine the number of segments formed outside the growth zone (Fig. 1d). Segments should be at least seven cells wide, and boundaries between segments should consist of a clear transition of the expression of the segmentation gene from a high to a low level, or vice versa, within five cells (similar to earlier definitions [6, 8]). Given that the tissue grows out to be 134 cells, of which nine form the head segment and five form the growth zone, the maximum number of segments that can be formed is 18. The number of well-formed segments (i.e. fulfilling the above requirements) determines an individual’s fitness. In addition, some penalties are applied. First, we require that at least one gene of each type is present in the genome; if this requirement is not met, the individual is not allowed to reproduce. Second, too-narrow segments are penalized. Third, small fitness penalties are used for gene and TFBS numbers in order to prevent excessive genome growth. Finally, when determining the number of segments, rather than considering the expression of the segmentation gene at the last time step of development, we average expression of the segmentation gene over the last 100 developmental steps. This averaging helps ensure temporally stable segmental patterning, as it will not reward oscillatory segmentation that fails to converge on a constant spatial pattern. To further ensure stability of the final developmental pattern, we apply an additional fitness penalty on the number of cells which have high variance in their gene expression over time, indicating pattern instability within these final 100 developmental steps. The fitness then becomes ${\mathrm{e}}^{{\mathrm{max}}(0,F)}-1$, where *F* is:

2 $$\begin{aligned} \begin{aligned} F&={\text {nr good segments}}\\&\quad - {\text {nr narrow segments}}\\&\quad - G*{\text {gene nr}}\\&\quad - T*{\text {TFBS nr}}\\&\quad - U*{\text {nr unstable cells}} \end{aligned} \end{aligned}$$

See Table 1 for parameter values.

**Fig. 3:**
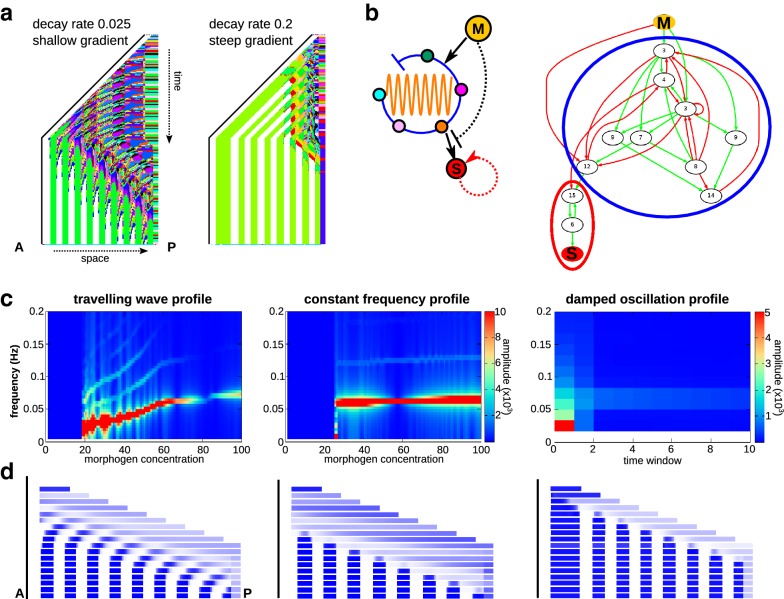
Summary of simulation results. **a** Examples of the resulting space–time plots from an individual at the end of a simulation. The posterior growth zone on the right is anchored and the other cells shift position when the tissue grows. The colour reflects the cell type, which is determined by the precise combination of expression levels of all genes within a cell. Note the regular alternation of gene expression in the posterior growth zone. **b** Left: a simplified representation of the gene regulatory networks that evolve in our simulations; right; an example of an evolved network (pruned, see "Methods"). The clock that generates gene expression oscillations is indicated in blue, the bistable switch in red. **c** Frequency profile of the segmentation gene (wave and constant frequency profiles) or the strongest oscillating gene (damped profile) in three simulations with a shallow gradient. **d** Snapshots of the tissue-level gene expression, corresponding to the profiles in **c** (blue is high expression, white is low). The anterior ends (indicated by the black bars) are aligned for greater clarity. The pictures are taken 12 steps apart

#### Evolution

*Initial conditions, mutations and simulations* The population is initialized with 50 identical individuals. The population resides on a lattice of size $30\times 30$, imposing an upper boundary of 900 individuals to the population size. The genome of the initial individuals contains a single copy of each gene, in randomized order and with an average of two TFBS of random type upstream. Individuals compete in a local $7\times 7$ neighbourhood for the opportunity to reproduce into an empty spot. As mentioned before, local competition is more computationally efficient than all-against-all fitness comparisons and better reflects the natural situation. An individual’s chance to reproduce is proportional to its fitness divided by the sum over the fitness values of itself and the other individuals neighbouring the empty position: $P_i=\frac{f_i}{\sum _{j=1}^{nb}f_j}$. Death occurs with a constant probability *d*, and individuals move on the lattice via Margolus diffusion (two diffusion steps, one of each partition, per update step).

Upon reproduction, the genome is mutated via duplications and deletions of TFBS and genes (including upstream TFBS), with a per-element probability (Fig. 1e). TFBS may also mutate their type (which gene product binds) and weight (activating or repressing), and new TFBS may appear de novo as an innovation. Gene duplication results in multiple genes of the same type that together determine the concentration of a single protein. Note that since we do not include mutations that change gene type, gene duplication cannot be followed by subsequent divergence. In order to simplify our model and decrease the number of different mutation rates in our simulations, we do not evolve maximum gene expression rates, protein decay rates or TF activation and deactivation thresholds (parameters *E*, *D* and *H* in Eq. 1) similar to the approach taken in [8].

### Analysis pipeline

It is highly non-trivial to derive the patterning strategy of an evolved network merely by looking at network architecture. Even for small networks evolved to the simple task of patterning a single stripe along the body axis, identical network architectures may lead to different patterning dynamics for different regulatory interaction strengths [30]. Additionally, patterning outcomes will depend on details of how transcription factor input is integrated, for example whether multiple activating transcription factors need to be simultaneously present (a logical AND gate), or rather that a single one suffices (a logical OR gate) to induce the downstream gene. Thus, to identify the patterning strategy, one needs to simulate the dynamics of gene expression resulting from the network, parameter settings and transcription factor integration. For small networks, it may still be feasible to determine the patterning strategy by examining the expression dynamics of individual genes; this strategy, however, will not provide a solution for larger networks evolved towards more complex patterning tasks, such as the one considered here. Previously, mostly individual case studies (selected from larger sets of evolutionary outcomes) were used to unravel the evolved developmental mechanism, analysing only a few network architectures and their gene expression dynamics in detail [6, 8, 10, 20, 31, 32]. However, if we aim to study the circumstances that drive evolution of complex oscillator networks and/or of sloped oscillatory frequency gradients, large numbers of simulation outcomes need to be assessed. Detailed manual analysis of each individual simulation outcome would be prohibitively slow. Furthermore, a different type of approach is needed to determine the nature of the evolved segmentation oscillator, i.e. whether it generates damped or persistent oscillations, and whether oscillation amplitude or period changes gradually or abruptly as a function of morphogen concentration. Therefore, we developed an automated analysis pipeline that can determine measures of network complexity and oscillatory frequency profiles for large numbers of simulations. This pipeline assesses for each individual simulation the size of the genome and complexity of the gene regulatory network: the genome is pruned beforehand to remove redundant elements and obtain the core network responsible for patterning. The evolved gene expression dynamics are assessed with Fourier analysis, to reveal the oscillatory dynamics at various points in the tissue.

#### Complexity analysis

Our pipeline starts by extracting from each simulation the genome of a single fit individual present in the population at the end of evolution. Because an evolved genome consists partly of redundant interactions, we first prune the genomes via a repeated process of trying to remove genes and binding sites in the genome, while keeping the final spatial expression pattern of the segmentation gene the same [8]. We will refer to these pruned genomes and networks as core genomes and networks, as they embody the essential core necessary to generate the segmentation pattern. To obtain measures for the complexity of the evolved networks, we determine genome size (number of genes and TFBS), the number of regulatory loops present in the network encoded by the genome, the size (nr of genes) of these loops and the number of positive and negative feedback loops. All measures are obtained for the core genomes and networks.

#### Fourier frequency profile analysis

Since the model incorporates posterior growth, we expect a significant part of the evolutionary runs to evolve sequential segmentation, where temporal gene expression oscillations are translated into a spatial segment pattern [10]. To determine the precise nature of the oscillations, we apply a fast Fourier transform (FFT, C library fftw3.h) to the gene expression dynamics and quantify how the amplitude and frequency of oscillations change as a function of morphogen concentration. Since each cell leaving the posterior growth zone experiences the same morphogen decay, such an analysis will reveal both the temporal oscillation dynamics of an individual cell and the spatial oscillation profile across the tissue at a single time point. This method will therefore allow us to determine whether, in case of persistent oscillations, a sloped frequency profile is present and kinematic oscillation waves are to be expected.

In principle, one could apply Fourier analysis directly to the gene expression dynamics of a cell as it leaves the growth zone and experiences morphogen decay. However, cells leaving the growth zone undergo only few oscillations in a short amount of time, and there are only a limited number of timepoints per individual morphogen concentration level. This makes it hard to extract the precise oscillatory dynamics as a function of morphogen concentration, especially when the morphogen decays rapidly. Furthermore, such an analysis would not be able to distinguish whether, at any given morphogen concentration, oscillations are stable or damped. Therefore, we decided to obtain longer time series of gene expression by running the evolved networks multiple times, each time with a different but constant morphogen concentration, using a linear set of concentration levels occurring along the morphogen gradient (Fig. 2a). This ensures that the same amount of data and detail is available for oscillators evolved under fast and slow morphogen decay.

After developing this series of gene expression dynamics for different morphogen concentrations, we apply a Fourier analysis for each individual gene for each of these different time series (Fig. 2a). Subsequently, we select the gene oscillating with the largest amplitude. For this gene, we then plot the frequency distributions (amplitude per frequency) for each morphogen concentration next to each other in a 2D heat map, creating the so-called frequency profile (Fig. 2a). We give examples of the resulting plots in Fig. 2c, first column. Note how the frequency of the oscillations may or may not change with the morphogen concentration. A side effect of using this Fourier analysis is that, in addition to detecting the frequency of the genetic oscillator as the dominant mode, it also detects one or more so-called eigenmodes of this frequency, as can be clearly seen in Fig. 2c, second row. These eigenmodes have no particular biological meaning.

We also investigate whether the frequency or the amplitude of oscillations changes within the growth zone, and whether oscillations are damped or persistent. To do so, we apply Fourier analysis to different subsections of the time series for the high morphogen concentration occurring in the growth zone (Fig. 2b). The procedure for making the frequency profile heat map remains the same, but now the *x*-axis represents developmental time rather than morphogen concentration. Examples can be found in Fig. 2c, second column.

#### Oscillator classification

To compare the evolutionary outcomes under different morphogen decay rates, gene expression noise levels and cell–cell signalling, we would like to classify the obtained frequency profiles into the three different categories illustrated in Fig. 2c. First, we distinguish between damped and persistent oscillators depending on the Fourier profile obtained from the growth zone. This is done by simple visual inspection of the profile, determining whether or not oscillations of nonzero amplitude persist throughout the time window. Next, within the category of persistent oscillators, we determine whether a frequency profile is constant across the morphogen gradient or rather has a sloped appearance, which is indicative of oscillations slowing down as morphogen levels decrease. This classification was formalized as follows: we measure the maximum oscillatory frequency occurring for the high morphogen concentrations in the posterior as well as the minimum frequency of the oscillations just prior to the ceasing of oscillations. Next, we determine the difference between these oscillation frequencies, indicating the extent of oscillator slowing across the morphogen gradient. We choose a particular threshold value for this frequency difference (0.02). For frequency differences larger than this threshold, we classify the oscillator as one with a sloped frequency profile, and for smaller frequency differences, we denote it as an oscillator with an approximately constant frequency profile.

## Results

### General evolutionary outcomes

We started with two sets of 60 simulations: one with a low and one with a high morphogen decay rate, leading to shallow and steep gradients, respectively. Nearly all simulations resulted in the evolution of a tissue pattern with ten or more segments, where ten is the threshold we use to classify a simulation as successful (59 of 60 simulations with a shallow gradient, 60 out of 60 simulations with a steep gradient were successful). Of these successful simulations, the maximum number of 18 segments evolved in ten shallow-gradient and 11 steep-gradient simulations. Typical space–time plots for both kinds of gradient are shown in Fig. 3a.

All mechanisms that evolved in our simulations use gene expression oscillations (a ’clock’) coupled to a bistable switch to generate segments sequentially, which is in line with our previous studies [8, 10]. Due to the nonlinearity of gene expression regulation in our model, a positive feedback on the segmentation gene allows for bistability to stably maintain either high or low expression of this gene. In contrast, negative feedback loops can generate the oscillations of the clock, provided that there is sufficient delay between upregulation and inhibition [33]. Because we did not include the evolution of protein decay rates or expression levels, in our model evolution generates the necessary delays by connecting a series of genes into a negative feedback loop. The networks evolved in our simulation typically contain multiple interconnected negative feedback loops, with one or more of them connected to the bistability motif.

Typically, one or more of the negative feedback loops are regulated by the morphogen (Fig. 3b). While the morphogen concentration is high, the network keeps oscillating between two regions that form the future basins of attraction of two unstable states formed by the bistable switch (Additional file 1: Fig. S1A). When morphogen concentrations drop, oscillations terminate, the two states become stable and the network converges to either the high- or low-segmentation gene expression state, depending on the phase of the cycle at which oscillations stopped. Thus, the bistability allows for a translation of oscillations into a stable segmented gene expression pattern. This structure is similar to the mechanisms that evolved in [8, 10], although the pruned networks tend to remain somewhat larger in our current model. Variations on this general theme do occur; for example, the inhibition by the morphogen may be indirect, or the segmentation gene and the genes in the positive feedback loop may be part of a negative feedback loop of the oscillator (Additional file 2: Fig. S2). Still, the overall mechanism always seems to use morphogen-dependent oscillations and translates them into a stable segmentation pattern with a bistable switch.

*Classifying evolved gene expression dynamics with Fourier analysis* We next assessed whether Fourier analysis would allow us to distinguish differences in the evolved gene expression dynamics of individuals from different simulations—despite the similar gene network structure. In short, we assessed how the frequency of oscillations changes when cells exit the growth zone. We found that the gene expression dynamics could be classified into roughly three different categories, which display qualitatively different frequency profiles (Fig. 3c). In the first column, the computed frequency profile clearly shows a slope, implying the occurrence of slower oscillations for lower morphogen concentrations (we call this a sloped frequency profile). In the snapshots of the segmentation gene expression that occurs during in silico development (Fig. 3d), we indeed see that every segment starts as a travelling wave from the posterior and becomes narrower and more strongly expressed as it arrives at the anterior. Thus, a sloped frequency profile corresponds to travelling waves across the tissue, much like those observed in vertebrate development.

In contrast, the individual used as an example in the middle column of Fig. 3c has a constant frequency profile, implying that oscillations have a constant frequency for a range of morphogen concentrations and then suddenly cease for lower morphogen concentrations (a constant frequency profile). The corresponding snapshots in Fig. 3d (centre) show that indeed, most of the tissue oscillates synchronously and that only the anterior end shows a minor deviation of these dynamics immediately prior to segment stabilization. Based on our frequency plot, we can deduce that in this small region, the cells are already in a non-oscillatory regime, converging towards one of the two stable states that allow for a segmented pattern. Note that this is different from the individual with travelling waves in the left column, where the anterior tissue that is out of sync with the posterior end is in a regime of sustained but slower oscillations.

Finally, in the right column of Fig. 3c, we display an individual whose frequency profile only shows oscillatory dynamics for the high morphogen concentrations that occur in the posterior growth zone. An analysis of the temporal dynamics of these growth zone oscillations (Fig. 3c) reveals that they are damped, reducing their amplitude over time (a damped frequency profile). This is confirmed by the snapshots of gene expression dynamics, which show a clear decrease in oscillation amplitude in the growth zone (Fig. 3d).

In all three cases illustrated above, there is a clear correspondence between the developmental gene expression dynamics as suggested by the computed Fourier frequency profile and the actual observed developmental dynamics. We therefore conclude that the Fourier frequency analysis is a useful tool for distinguishing differences in the oscillatory dynamics produced by evolved networks. Note that while the above examples are easily distinguishable, clear-cut cases, not all evolved mechanisms generate frequency profiles that are easy to interpret or fall into these three clear categories. Some profiles have a very modest slope; in other cases, oscillations extend beyond the growth zone but for only a limited part of the entire morphogen concentration range; and in yet other cases, oscillations may be damped for the high morphogen concentrations in the growth zone yet persistent for a range of lower concentrations (Additional file 3: Fig. S3). Still, also for these more complicated cases, the frequency profile reliably reflects the actual oscillatory developmental dynamics.

### Shallow gradients promote sustained oscillations and travelling waves

**Fig. 4:**
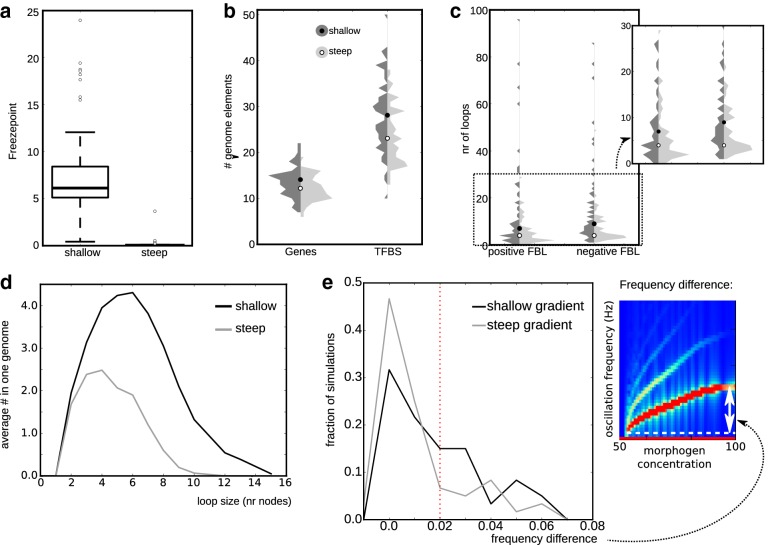
Comparison of genome, network and oscillatory dynamics properties. **a** Boxplot of the morphogen level at which individuals reach a stable expression (after the transition from the oscillatory to the non-oscillatory regime). **b** Violin plots (vertical histogram) of the number of genes and transcription factor binding sites (TFBS) in the pruned genomes of shallow-gradient (dark) and steep-gradient (light) simulations. Dots indicate the median value. Mann–Whitney *U* test between shallow and steep: genes, $p=0.006$; TFBS, $p=0.0007$. After removing the 14 largest genomes from both sets: genes, $p=0.003$; TFBS, $p=0.0001$ (corrected for ties with jitter). **c** Violin plots of the number of positive and negative feedback loops in the pruned networks of the shallow- and steep-gradient simulations. (MW test: pos.FBL, $p=0.005$; neg.FBL, $p=0.0003$. After removing 14 genomes with most loops: pos.FBL, $p=0.008$; neg.FBL, $p=0.0001$). **d** Histogram of the number of loops (FFL and FBL) of a certain size. All histograms of individual simulations have been summed for this average histogram. **e** Histogram displaying for all successful individuals their frequency difference between oscillations in the growth zone and at the end of the profile, before sustained oscillations cease. (see indication in the profile on the left: a nice example of a strongly sloped frequency profile with a large difference). Profiles to the right of the red line are classified as “sloped” in Table 2. Note that the damped oscillators are grouped in the bin with 0.0 frequency difference. Bin size: 0.01

To test how the length and slope of the morphogen gradient influence the evolution of segmentation, we next compared segmentation mechanisms evolved under high versus low morphogen decay rates. The two space–time plots shown earlier in Fig. 3a illustrate that the spatio-temporal transient during which cells are outside the growth zone but have not yet formed a segment, is considerably longer for shallow morphogen gradients than for steep gradients. We measured at which morphogen concentration oscillations cease and a stable stripe pattern is formed, the so-called freeze point, and found that under a shallow gradient, higher freeze points evolve (Fig. 4a). However, the position of this higher freeze point in the tissues with a shallow gradient is still further away from the growth zone than the position of the near-zero freeze point in the tissues with a steep gradient. The higher freeze point therefore only partially compensates for the longer time and distance required for morphogen decay. The question is whether this spatio-temporally extended transient—and the accompanying freeze point shift—has evolutionary consequences in terms of network complexity and the types of oscillatory dynamics that evolve.

**Table 2:** Prevalence of frequency profiles

Noise level	Gradient slope	Successful sims. (out of 60)	Damped profiles (nr and fraction of total)		Constant freq. profiles		Sloped profiles		Not classified	
None	Shallow	59	3	0.05	36	0.61	16	0.27	4	0.07
	Steep	60	14	0.23	36	0.60	7	0.12	3	0.05
Low	Shallow	51	1	0.02	20	0.39	20	0.39	10	0.20
	Steep	60	5	0.08	44	0.73	8	0.13	3	0.05
Medium	Shallow	53	2	0.04	14	0.26	31	0.58	6	0.11
	Steep	60	1	0.02	27	0.45	21	0.35	11	0.18
High	Shallow	33	0		8	0.24	22	0.67	3	0.09
	Steep	58	5	0.09	30	0.58	13	0.22	10	0.17

To investigate this, we deployed our analysis pipeline to dissect genome and network complexity and details of the oscillation dynamics. We found that under shallow gradients, individuals evolve that have somewhat larger core genomes and networks (a small but significant difference), especially because of a larger number of TFBS (Fig. 4b). The networks evolved under shallow gradients also contain significantly more feedback loops, in particular the negative FBLs needed to construct an oscillator (Fig. 4c), and these loops tend to be larger (Fig. 4d). The variability between individual evolutionary trajectories with a shallow gradient is large: the increase in average loop number and size for the simulations with a shallow gradient is exacerbated by a subset of 14 simulations (out of a total of 59) which have more than 20 negative feedback loops. These simulations also have the largest genomes (Additional file 4: Fig. S4). Still, differences in genome size and feedback loops remained significant when we compared the two sets after removing the 14 simulations from both (see legend Fig. 4).

When we classify the oscillatory dynamics of all simulations into the three broad categories of Fig. 3, the simulation set with a shallow gradient has a lower fraction of profiles with damped oscillations (shallow: 0.05 vs. steep: 0.23) and a higher fraction of sloped frequency profiles (0.27 vs. 0.12, Table 2), while the two sets contain a similar number of simulations with a constant frequency profile (0.61 vs. 0.60). To test the robustness of these results, we also measured the frequency difference within a profile (Fig. 4e), rather than categorizing the profiles using somewhat-arbitrary cut-offs to distinguish sloped from constant profiles. The distribution of these frequency differences makes it clear that not only do shallower gradients more often lead to the evolution of a sloped profile, but they also tend to evolve a slightly higher frequency difference across their profile (Fig. 4e).

### Gradient steepness influences evolutionary innovation speed

**Fig. 5:**
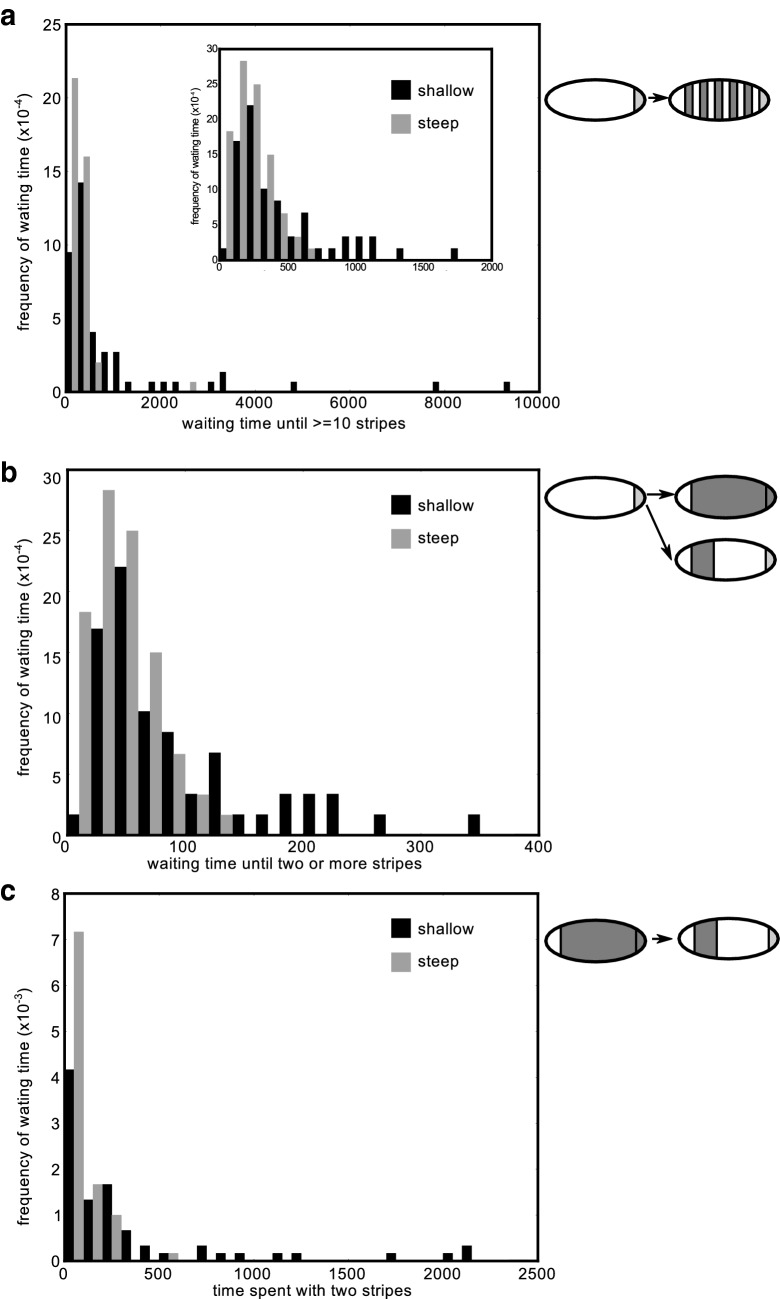
A shallow gradient takes longer to find a solution. **a** Histogram of the number of generations it took for simulations to make ten or more stripes. Bin $\hbox {size}=100$. **b** The waiting time until individuals with two or more stripes appear in the simulation. Bin $\hbox {size}=25$. **c** The number of generations each simulation spent with only two stripes (see space–time plot). Note that the first bin includes those individuals which immediately find more than two stripes. Bin $\hbox {size}=50$

We established that the steepness of the morphogen gradient influences both the type of oscillations that evolves and the network that generates these oscillations. Next, we investigated whether this difference in final evolutionary outcome is reflected by differences in the evolutionary trajectories leading up to these outcomes. We find that under a steep gradient, individuals with more than ten segments arise very early in evolution (Fig. 5a). In contrast, with a shallow gradient, the evolution of individuals with ten or more segments frequently required a much longer evolutionary time span. Much of this time, these evolutionary trajectories are either searching for or stuck in a primitive, two-segment stage, where the entire tissue that is generated by the growth zone expresses the segmentation gene while the head does not (Fig. 5b, c). These data indicate that it can be considerably harder for evolution to discover a segmentation pattern under a shallow gradient.

To further investigate this difference, we removed the “head” from the initial tissue (see Fig. 1c). As discussed in the Methods section, the head region is the part of the tissue in which the morphogen gradient is absent and no gene expression occurs. As a segment boundary is defined as the transition from low to high expression of the segmentation gene or vice versa, simply expressing the segmentation gene in the non-head part of the tissue thus suffices to generate the first segment. Removing the head region will make it harder for evolution to discover the first segment and may therefore in some cases make it impossible to evolve segments. The rate of success of evolutionary simulations indeed decreases significantly in the absence of a head region and considerably more so for shallow than steep morphogen gradients. Only 28 out of 60 simulations find a solution for a shallow gradient, while 51 out of 60 simulations evolve a segmented pattern with at least ten segments for a steep gradient. This further supports our observation that a segmented body pattern evolves more easily for steep morphogen gradients.

### Evolved segmentation mechanisms adapt easily to a different morphogen gradient

Having established that both final properties and evolutionary trajectories differ for segmentation mechanisms evolved under shallow or steep gradients, we next asked whether these differences are functionally relevant. To assess this, we extracted successful individuals evolved under a steep or shallow morphogen gradient and let them continue evolution in the presence of a morphogen gradient of the opposite steepness.

**Fig. 6:**
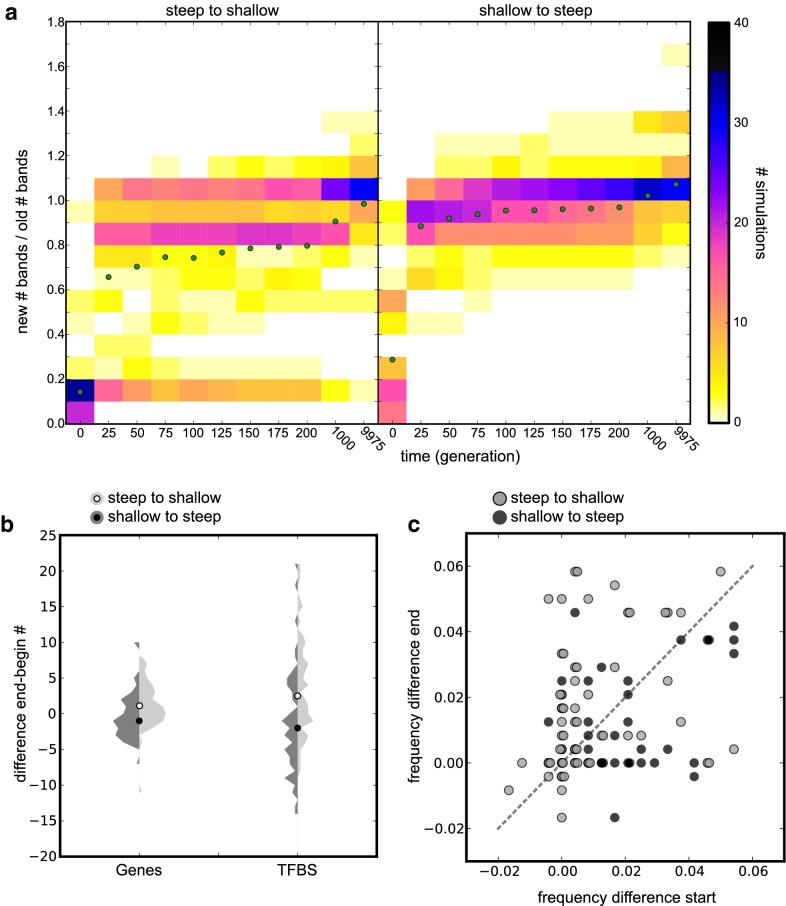
Switching to another decay rate reveals functionality of evolved differences. **a** Heat map of the number of segments (ratio original number of the transplanted individual/current nr of segments maximum fit individual) after switching the decay rate of all individuals. Dots indicate average ratio. **b** Violin plot of the difference in the number of genes and TFBS in the pruned genome between the start and end of the simulation. **c** Scatterplot with the frequency difference (see Fig. 4d) of the Fourier profile at the start and the end of the decay-switch run. Light is from steep to shallow, dark from shallow to steep

For a transition from a shallow to a steep morphogen gradient, 22 out of 59 simulations are immediately able to generate more than three segments (Fig. 6a). In contrast, for the transition from a steep to a shallow gradient, only six out of 60 simulations can directly generate more than three segments (Fig. 6a). Still, in both cases evolution generally needs fewer than 30 generations to come to a new solution with a similar number of segments as before the transition. For the steep to shallow transition, three simulations needed more than 1000 time steps to restore their prior segmentation pattern.

We conclude that a segmentation strategy evolved under one type of morphogen gradient is not automatically fully functional under the other type of morphogen gradient but requires evolutionary adaptation. Although this evolutionary adaptation occurs rapidly and readily, the need for it suggests that functional differences exist between segmentation mechanisms evolved under different morphogen gradient types. To investigate this, we looked at the difference in (pruned) genome size between the original individuals and an individuals at the end of the evolutionary transition simulation. We observe that for a transition from a shallow to a steep gradient, genome size is more likely to decrease, while for a transition from a steep to a shallow gradient, genome size is more likely to increase (Fig. 6b). Although the observed differences are small, they are in line with the differences in genome size we showed in Fig. 4.

Additionally, in Fig. 6c, we illustrate that the frequency profile also changes in accordance with our earlier results. For the evolutionary transition from a shallow to a steep gradient, the slope of the frequency profile is slightly more likely to decrease (27 decrease, 20 increase) and the number of damped oscillators increases (from 4 to 18). For the opposite evolutionary transition, the slope of the frequency profile is more likely to increase (32 increase, 19 decrease), and the number of damped oscillators decreases (16–7). Together this further supports the idea that differences between segmentation mechanisms evolved under shallow and steep gradients are functionally relevant.

Finally, the results of our transition experiments also imply that the evolutionary transition from shallow to steep is easier than that from steep to shallow. This agrees with our earlier findings on the difference in speed with which segmentation patterns evolve under shallow and steep gradients.

### Gene expression noise promotes sustained oscillations and travelling waves

Next, we aimed to find the functional differences between the types of segmentation mechanism evolving under steep or shallow morphogen gradients. We focused on the difference in the number of damped and travelling wave oscillators that evolve for steep and shallow morphogen gradients.

In case of persistent oscillators, gene expression dynamics follow a stable limit cycle spanning the basins of attraction of the future two stable segmentation states (Additional file 1: Fig. S1A). Persistent oscillations thus allow a stable memorization of the initial oscillation phase at the cell’s birth, right until the moment morphogen levels drop and the phase is translated into one of two segmentation states. In contrast, for damped oscillations, the gene expression dynamics are spiralling inward to the equilibrium inside the unstable limit cycle, which necessarily resides in only one of the basins of attraction of the segmentation states (Additional file 1: Fig. S1B). This causes cells to gradually lose their memory of their original oscillation phase, ultimately causing convergence to a single differentiated state irrespective of initial phase. We hypothesize that steep morphogen gradients suffer less from this memory loss as segmentation occurs rapidly, when damping has only just begun, and that this explains the higher likelihood of damped oscillators evolving under these conditions. Following this logic, we speculate that adding noise on gene expression could increase the sensitivity to phase memory loss: it might bring the cell faster to the single stable state by accident. Thus, we expect that noise decreases the fraction of simulations in which damped oscillators evolve, especially for steep gradient where damped oscillators are common.

Under shallow gradients instead, sloped frequency profiles and travelling waves commonly evolve while damped oscillators are rare. If we assume that there is no inherent difference in functionality between having a constant or a sloped frequency profile, the higher number of sloped profiles could simply be due to the more general need for sustained oscillations when the gradient is shallow. In that case, a sloped profile represents just one of two ways of achieving persistent oscillations. On the other hand, if a sloped frequency profile were to have any additional functionality, such as its suggested larger robustness [23], it may have more space and time to exert this functionality under a shallow, more spread out morphogen gradient. If this is the case, increasing selection for robustness should increase the likelihood of evolving segmentation mechanisms with travelling waves under a shallow gradient.

**Fig. 7:**
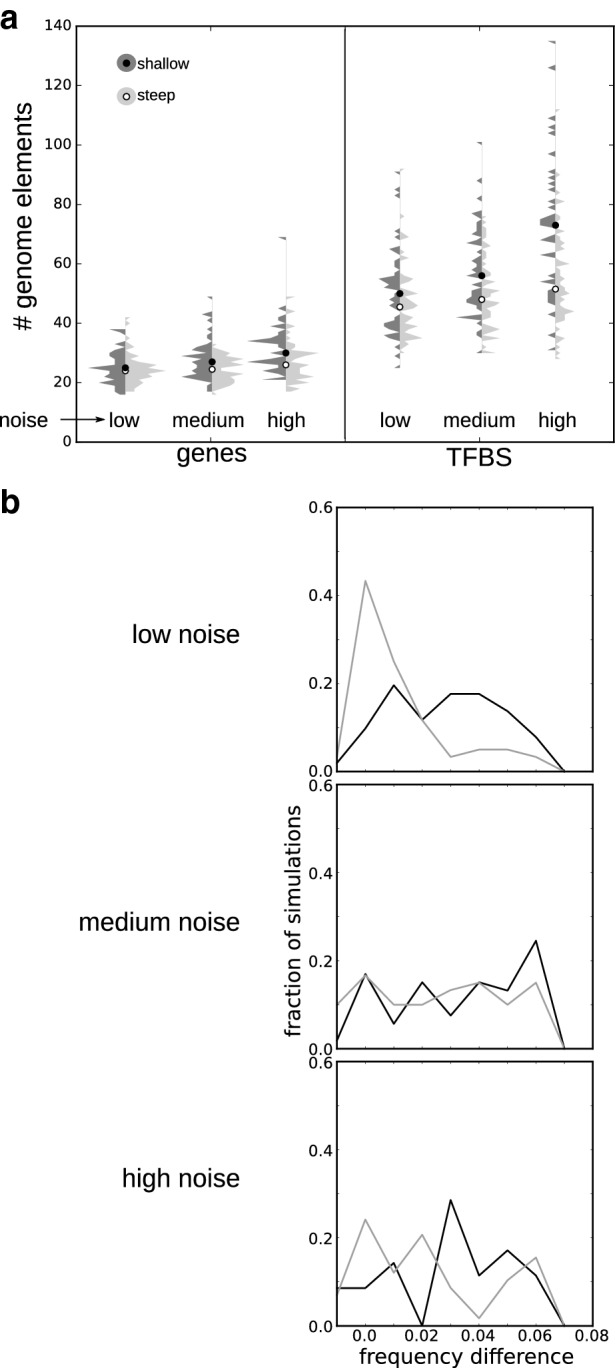
Genome size and oscillatory dynamics for different levels of gene expression noise. **a** Violin plots (vertical histogram) of the number of genes and transcription factor binding sites (TFBS) in the pruned genomes of shallow-gradient (dark) and steep-gradient (light) simulations. **b** Histogram of the frequency difference between oscillations in the growth zone and at the end of the profile

To test the above ideas, we added different levels of gene expression noise to our model, thereby inducing implicit selection for robustness. We found that the higher the noise, the lower the number of successful simulations; especially, the simulations with a shallow gradient were affected (Table 2). Furthermore, with higher noise, the size of the evolved genomes increases, mostly due to an increase in the number of TFBS, and again particularly noticeable for shallow gradients. These facts suggest that gene expression noise combined with a shallow morphogen gradient requires a more complex segmentation mechanism (Fig. 7a).

We find that adding noise greatly increases the fraction of simulations with a steep gradient that yield persistent oscillations (Table 2). This confirms our hypothesis that damped oscillators are only tolerated if limited memorization of oscillator phase is required. Strikingly, for simulations with a shallow gradient, all levels of gene expression noise yield an increase in the fraction of sloped frequency profiles that evolve (Fig. 7b). For medium and high noise levels, the fraction of sloped frequency profiles also increases in simulations with steep gradients. Together this confirms the hypothesis that a sloped profile increases robustness against noise.

**Fig. 8:**
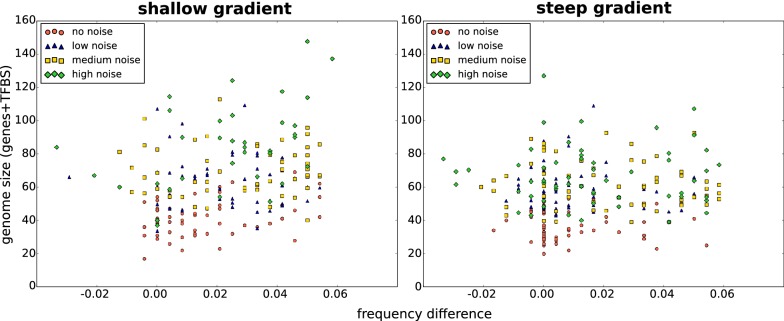
Relation between genome size and frequency difference. The *x*-axis represents the difference in oscillation frequency between the growth zone and the point before oscillations cease (see also Fig. 4d). The *y*-axis shows genome size as the sum of # genes and TFBS. No clear correlation between genome size and frequency difference is apparent. See Additional file 6: Fig. S6 for separate scatterplots for each condition (noise level and gradient steepness)

Given that shallow gradients and noise enhance both genome size and the occurrence of sloped frequency profiles, we investigated the correlation between these two properties: perhaps the increase in genome size observed with higher noise reflects the requirement for travelling waves. In Fig. 8, we plot the frequency difference (a measure for the slopedness of the frequency profile) against the genome size of simulations with different noise levels. (See also Additional file 5: Fig. S5 for plots separated by simulation condition, and Additional file 6: Fig. S6 for correlation with nr of loops.) From this, we conclude that no correlation exists between these two properties for individual evolutionary outcomes and that they likely evolved independently.

## Discussion

Segmentation is a major evolutionary innovation exhibited by the vertebrate, arthropod and annelid clades [1, 2]. In vertebrates, annelids and most arthropods, segments are generated in an anterior–posterior sequence and originate from a localized posterior growth zone. In vertebrates and arthropods, this sequential segmentation arises from oscillatory gene expression in the posterior growth zone, where morphogen levels are high. As cells are pushed out of this zone and morphogen levels drop, oscillations cease and a temporally stable gene expression pattern arises that prepatterns the segments. Despite this common clock-and-wavefront mechanism, intriguing species differences exist. While vertebrates and, for example, the arthropod Strigamia appear to have a long unsegmented zone and extensive kinematic waves, the cephalochordate Amphioxus and the beetle Tribolium appear to have shorter unsegmented regions and no or less extensive travelling of gene expression waves [4, 24]. Additionally, in Amphioxus and Tribolium, the oscillator clock appears to be less complex than in vertebrates and other arthropods [25, 26], although this may reflect merely a lack of data. It is currently unclear to what extent size of the posterior growth zone, oscillator slowing and oscillator complexity are related. Additionally, while both oscillator slowing and oscillator complexity have been suggested to contribute to developmental robustness, this has not been explicitly investigated.

To investigate these matters, we extended previous evo-devo models for the evolution of body axis segmentation by incorporating growth from a posterior growth zone, with a posteriorly expressed morphogen that forms a gradient through decay. We have previously shown how this biases evolution towards oscillatory sequential segmentation [10]. In addition, we developed an analysis pipeline that allows us to compute parameters describing network complexity and oscillatory dynamics. With this, we investigated the effect of different morphogen decay rates, resulting in differently sloped morphogen gradients and hence differently sized unsegmented zones. In addition, we also investigated the influence of gene expression noise, resulting in different levels of selection for robustness. We showed that in our new model, different types of oscillators can evolve, with either damped oscillators or oscillators with a constant period frequently evolving. In a subset of simulations, we also observed the spontaneous evolution of oscillators with a sloped frequency profile resulting in a slowing down of oscillations and generation of travelling waves towards the anterior [14, 34], similar to those seen during, for example, vertebrate somitogenesis or Strigamia segmentation. Furthermore, for these sloped frequency profiles, we find that oscillation frequencies typically decrease by 50–60% before oscillations cease rather than decreasing all the way to zero, in agreement with experimental measurements of vertebrate somitogenesis [35].

We found that a steep morphogen gradient more often leads to the evolution of a damped oscillator. Under a shallow morphogen gradient, cells go through a prolonged transient before oscillations cease, so we hypothesize that sustained oscillations may be needed to maintain a robust dynamic memory of the oscillator phase with which the cell left the growth zone. We also show that in the presence of gene expression noise, the number of evolved persistent oscillators increases for steep morphogen gradients, supporting the notion that persistent oscillators contribute to robust patterning.

In addition to differences in the occurrence of damped oscillators, shallow gradients also more often yield a sloped frequency profile. The likelihood of evolving travelling waves increases in the presence of gene expression noise, particularly for shallow gradients but also for steep gradients when noise levels are high. Our study thus confirms the hypothesis that sloped frequency gradients enhance the robustness of sequential segmentation. As to the mechanism of this enhanced robustness, we speculate that the slowing down of oscillations causes cell dynamics to spend more time inside the basins of attraction of the two segmentation states and relatively less time “in limbo” in between these two basins where it is less clear what to do when oscillations stop. As a consequence, the vulnerability to noise decreases.

Finally, we found that genomes evolved under a shallow gradient tend to be larger and that networks have more and larger feedback loops, with noise contributing to this effect. In a switch experiment, we let evolved individuals continue evolution in the presence of a gradient of the opposite steepness. The results from these simulations suggest that the observed differences in genome size and frequency profiles, while small, are functionally significant, since simulations switched from a shallow to a steep morphogen gradient tend to decrease their genome size and slope of the frequency profile, and vice versa.

Our results suggest a potentially important role for morphogen gradient length in causing the differences in segmentation processes found between species within both the arthropod and chordate clades. For instance, in both vertebrates and the centipede Strigamia, segmentation is preceded by a long spatio-temporal transient that is accompanied by extensive kinematic waves of gene expression [11, 18, 19, 35–37]. This is reminiscent of the outcomes we observed for a shallow morphogen gradient. Additionally, at least for vertebrates, the segmentation network is known to be highly complex and consists of an entanglement of three signalling pathways: FGF, Wnt and Notch [16], again similar to simulation outcomes under a shallow morphogen gradient.

In contrast, the cephalochordate Amphioxus lays down its segments very close to the posterior growth zone, and no travelling waves have (thus far) been observed. Additionally, the FGF pathway does not seem to be involved in segmentation [24–26], suggesting a simpler oscillator network architecture. Based on the currently available data, it thus appears that Amphioxus segmentation more closely resembles the in silico mechanisms evolved under a steep gradient. On a similar note, in the beetle *Tribolium*, segment formation occurs relatively close to the posterior growth region, and both the travelled distance and contraction of kinematic waves are modest, indicating only a slightly sloped frequency profile [4]. Additionally, the currently available data suggest a relatively simple oscillator network [27].

Importantly, our switch experiments demonstrate that evolution easily adapts a short gradient mechanism into a long gradient mechanism and vice versa. This supports the generally accepted notion that at least within a single clade segmentation evolved once and that within-clade differences arose through subsequent divergence of the segmentation mechanism. Based on our finding that simulations with steep and shallow gradients differ in the ease with which segments evolve, we speculate that the initial evolution of segmentation within a clade was of the steep-gradient type.

Recent studies have suggested that network complexity may reflect the need for two distinct oscillators, one with a constant frequency and one slowing down according to a decreasing frequency profile, with the resulting phase difference patterning somite boundaries and polarity [20, 36]. Intriguingly, in our simulations we did not observe a clear correlation between the evolution of high network complexity and travelling waves, despite the fact that the evolution of both these properties becomes more likely under shallow morphogen gradients and gene expression noise. These results demonstrate that (further) network complexity is not required for a sloped frequency profile. Instead, we speculate that network complexity is required for oscillator robustness and persistence. Together this suggests that network complexity and travelling waves could have evolved separately rather than simultaneously and may in fact play subtly differing roles.

Obviously, in order to simulate developmental processes in many individuals and over many generations in a computationally tractable manner, the developmental process in our model was highly simplified. Important simplifications are the restriction to a one-dimensional tissue architecture and the absence of cell motility. These would be highly interesting extensions for future studies, as two-dimensional tissue architecture likely increases the impact of gene expression and morphogen gradient noise on segment formation, while cell motility instead has been shown to contribute to patterning robustness [38]. Importantly, although simplified, our current model did contain the necessary ingredients that enabled us to investigate the evolution of kinematic waves, in contrast to earlier models in which morphogen gradient shapes were superimposed and kept constant.

### Conclusions

In summary, we have shown that gradient slope and length influence the evolution of travelling waves in segmentation. First, we showed that shallow gradients lead to the evolution of slightly larger genomes and networks with more and larger loops as compared to steep gradients, and more often to persistent oscillations with travelling waves. We also showed that these differences are likely to be functional. Finally, we showed that gene expression noise increases the likelihood of evolving persistent oscillators, and, especially in the presence of shallow gradients, of evolving travelling waves. We therefore propose that gradient length and noise may play a role in creating the differences observed both between species within the chordate and arthropod clades.

## Additional files

**Additional file 1.** Networks with persistent and damped oscillations have different origins. A) Persistent oscillations are the result of a stable limit cycle around an unstable equilibrium (open blue dot). As long as conditions (e.g. morphogen concentration) stay constant, these oscillations continue indefinitely. When the morphogen concentration decreases, the system will reach either of the two stable states (red dots), depending on the basin of attraction (red zones) in which it finds itself. B) Damped oscillations are caused by a stable spiral. Even if all else stays constant, the oscillations lose amplitude over time, and the system will end up with fixed gene expression. Such a system “loses” the memory of the oscillations and thus of the phase with which it started.

**Additional file 2.** Networks with different structures. A) In this network, the genes constituting the bistable switch are also part of the oscillator. B) The segmentation gene can itself also be part of the oscillator. In this case, the genes responsible for generating a bistable switch are hard to identify, also due to the size of the network. Both networks are pruned, with the requirement that the number of segments should stay the same.

**Additional file 3.** Examples of profiles that are harder to classify.

**Additional file 4.** Larger genomes generate networks with more loops. Scatterplot of the number of loops in the network versus genome size. The two are clearly correlated, but note that particularly simulations with a shallow gradient (red dots) lead to larger genomes and networks with more loops.

**Additional file 5.** The type of frequency profile is not correlated with genome size. Scatterplots of the posterior to anterior frequency difference in the profile versus genome size, separated by simulation condition (gradient steepness and noise level).

**Additional file 6.** The type of frequency profile is not correlated with the number of loops. Scatterplot of the posterior to anterior frequency difference in the profile versus the number of loops in the network.

## Abbreviations

TF: transcription factor
TFBS: transcription factor binding site
FBL: feedback loop
