# Supplementary figures and images for: Around the clock: gradient shape and noise impact the evolution of oscillatory segmentation dynamics
Source: EvoDevo. 2018 Dec 10;9:24. doi: 10.1186/s13227-018-0113-2 (PMC6288972; doi:10.1186/s13227-018-0113-2)

## Sustained oscillations

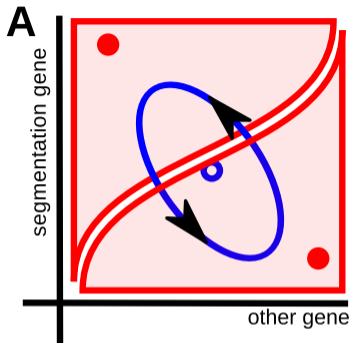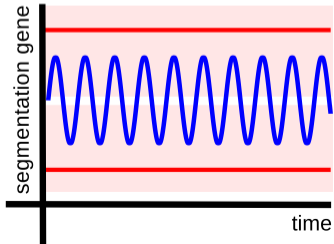

## Damped oscillations

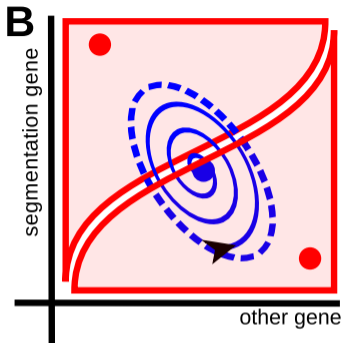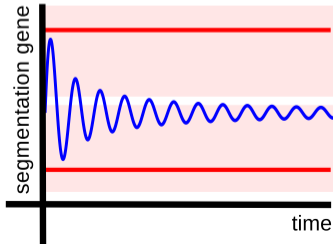

Supplement: Supplementary file 1 — Additional file 1. Networks with persistent and damped oscillations have different origins. A) Persistent oscillations are the result of a stable limit cycle around an unstable equilibrium (open blue dot). As long as conditions (e.g. morphogen concentration) stay constant, these oscillations continue indefinitely. When the morphogen concentration decreases, the system will reach either of the two stable states (red dots), depending on the basin of attraction (red zones) in which it finds itself. B) Damped oscillations are caused by a stable spiral. Even if all else stays constant, the oscillations lose amplitude over time, and the system will end up with fixed gene expression. Such a system “loses” the memory of the oscillations and thus of the phase with which it started. [file 13227_2018_113_MOESM1_ESM.pdf]

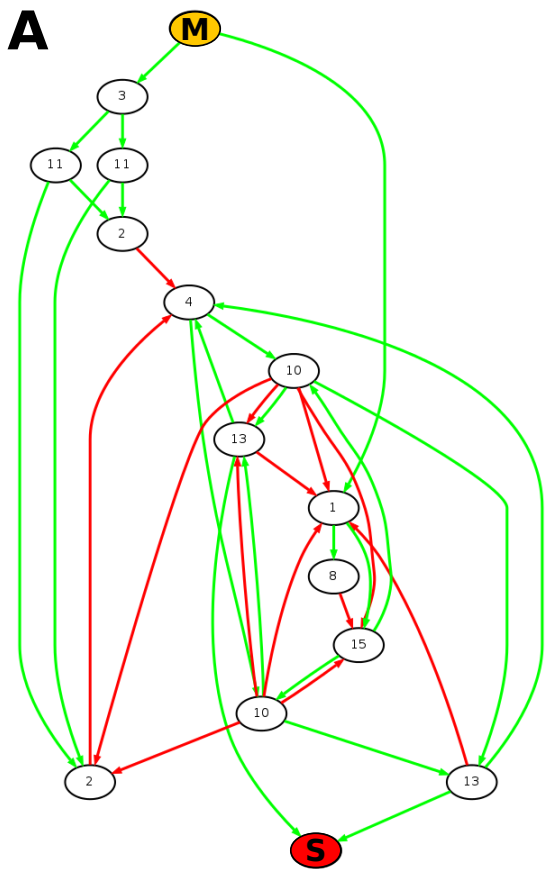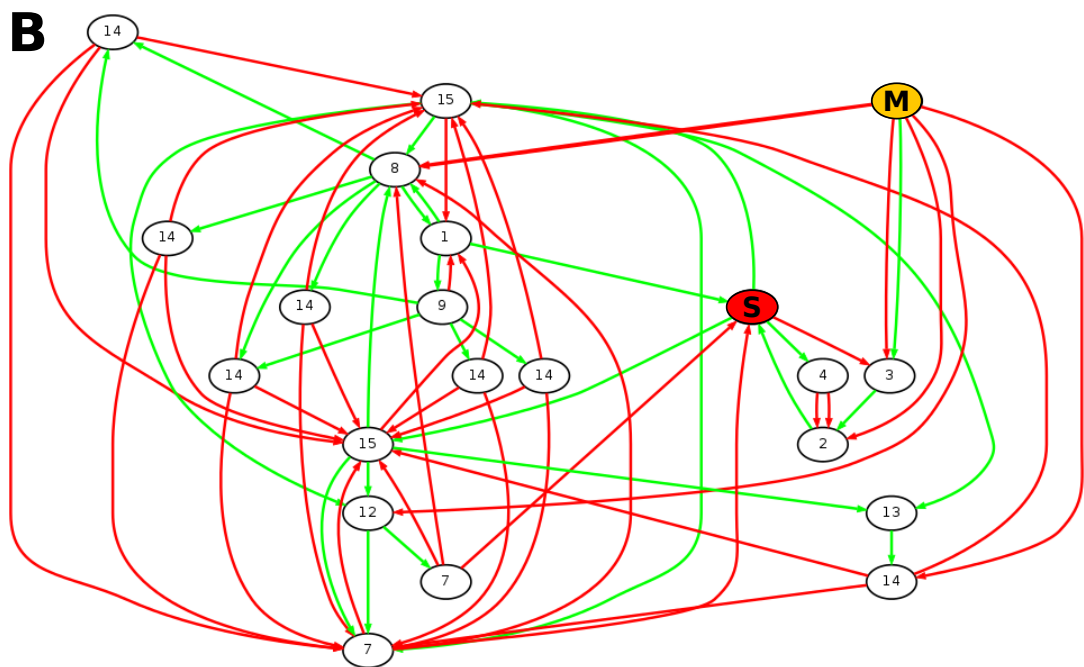

Supplement: Supplementary file 2 — Additional file 2. Networks with different structures. A) In this network, the genes constituting the bistable switch are also part of the oscillator. B) The segmentation gene can itself also be part of the oscillator. In this case, the genes responsible for generating a bistable switch are hard to identify, also due to the size of the network. Both networks are pruned, with the requirement that the number of segments should stay the same. [file 13227_2018_113_MOESM2_ESM.pdf]

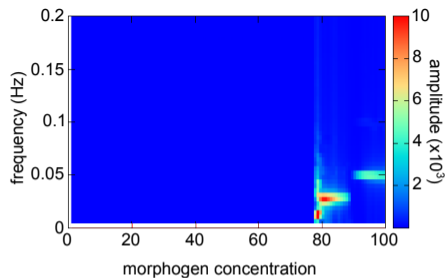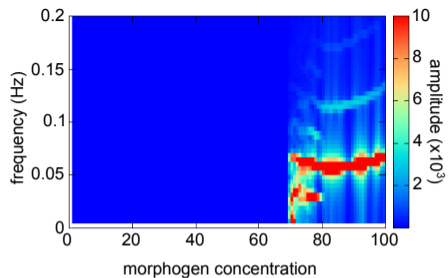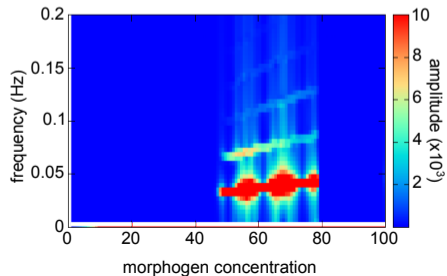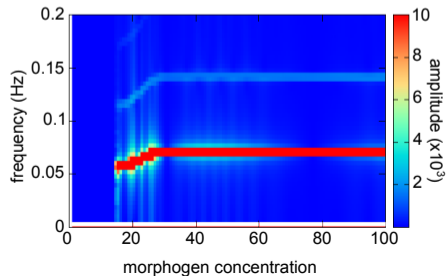

Supplement: Supplementary file 3 — Additional file 3. Examples of profiles that are harder to classify. [file 13227_2018_113_MOESM3_ESM.pdf]

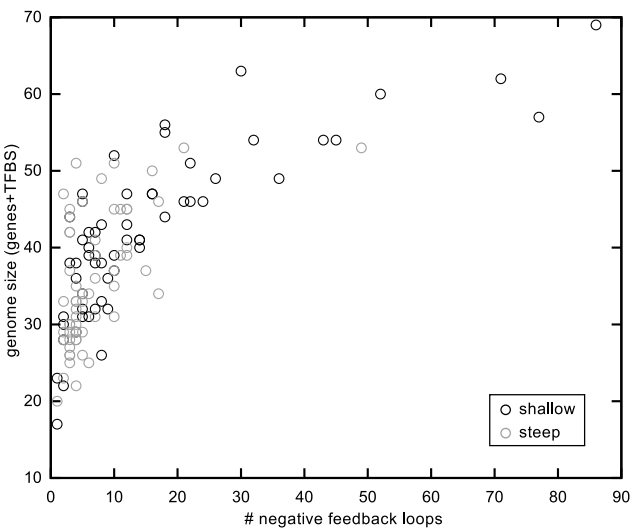

Supplement: Supplementary file 4 — Additional file 4. Larger genomes generate networks with more loops. Scatterplot of the number of loops in the network versus genome size. The two are clearly correlated, but note that particularly simulations with a shallow gradient (red dots) lead to larger genomes and networks with more loops. [file 13227_2018_113_MOESM4_ESM.pdf]

## shallow gradient

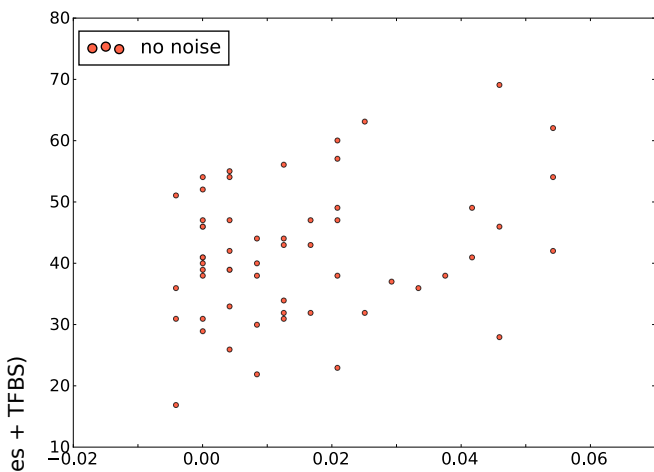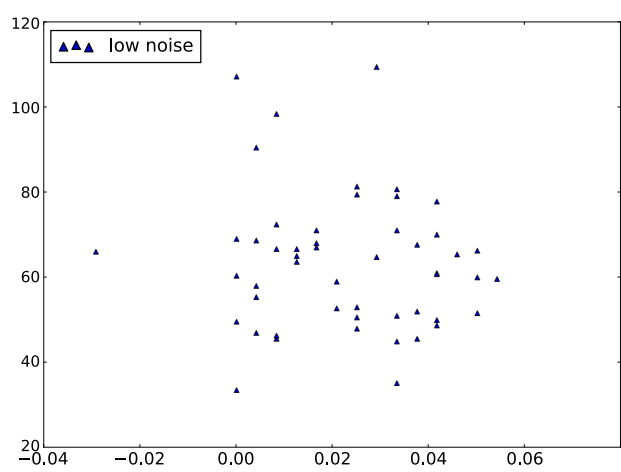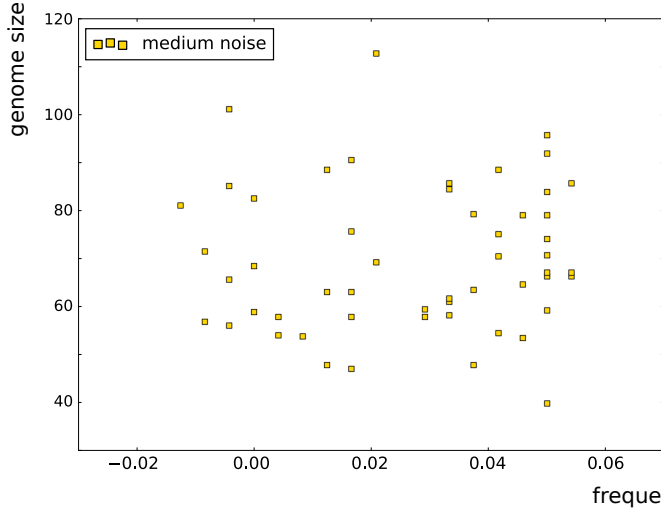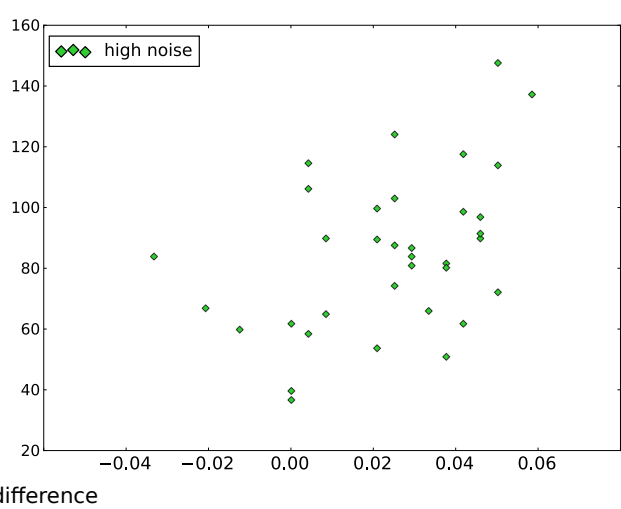

## steep gradient

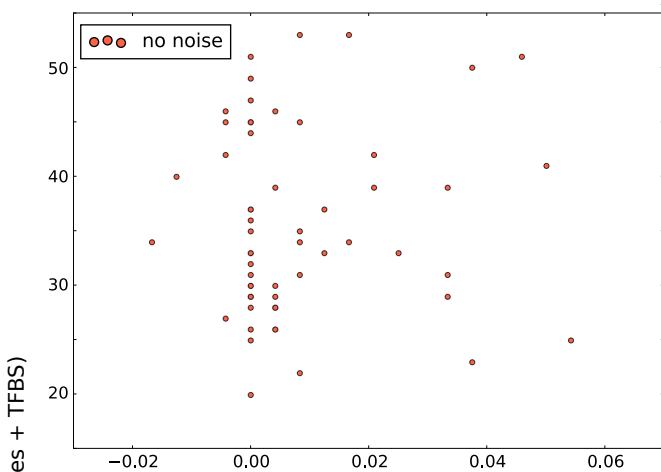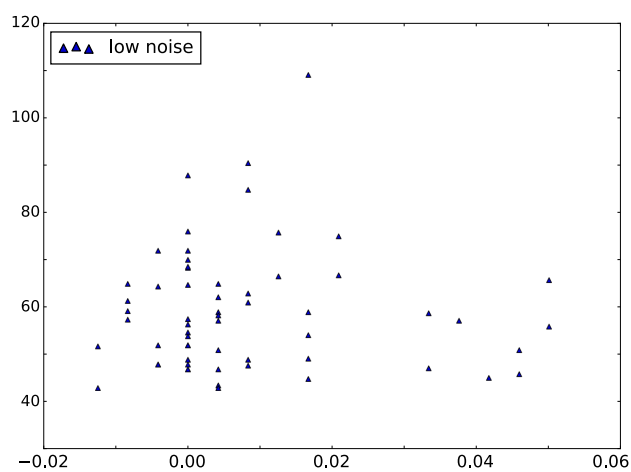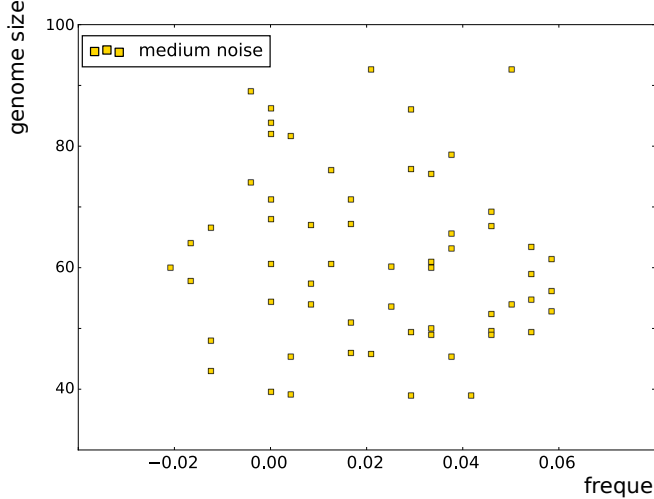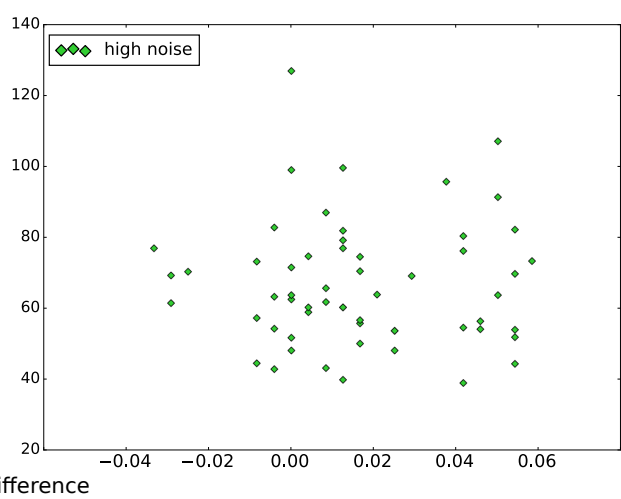

Supplement: Supplementary file 5 — Additional file 5. The type of frequency profile is not correlated with genome size. Scatterplots of the posterior to anterior frequency difference in the profile versus genome size, separated by simulation condition (gradient steepness and noise level). [file 13227_2018_113_MOESM5_ESM.pdf]

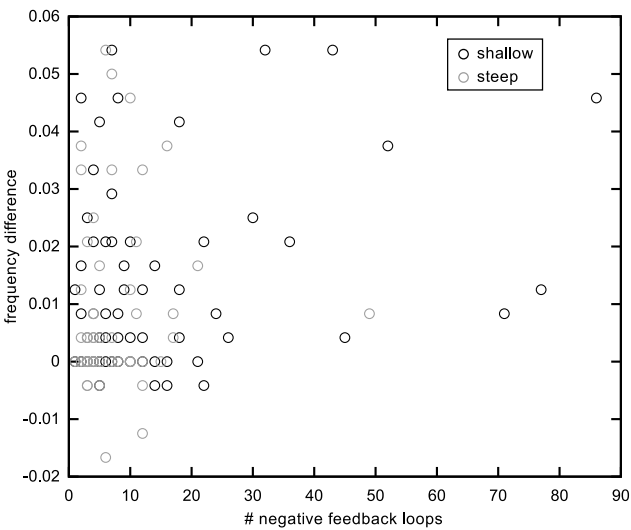

Supplement: Supplementary file 6 — Additional file 6. The type of frequency profile is not correlated with the number of loops. Scatterplot of the posterior to anterior frequency difference in the profile versus the number of loops in the network. [file 13227_2018_113_MOESM6_ESM.pdf]
